# Can CD200R1 Agonists Slow the Progression of Osteoarthritis Secondary to Injury?

**DOI:** 10.3389/fimmu.2022.836837

**Published:** 2022-03-14

**Authors:** Kathak Vachhani, Aaron Prodeus, Sayaka Nakamura, Jason S. Rockel, Adam Hopfgartner, Mohit Kapoor, Jean Gariépy, Cari Whyne, Diane Nam

**Affiliations:** ^1^ Institute of Biomedical Engineering, University of Toronto, Toronto, ON, Canada; ^2^ Sunnybrook Research Institute, Toronto, ON, Canada; ^3^ Department of Medical Biophysics, University of Toronto, Toronto, ON, Canada; ^4^ Division of Orthopaedics, Osteoarthritis Research Program, Schroeder Arthritis Institute, University Health Network, Toronto, ON, Canada; ^5^ Krembil Research Institute, University Health Network, Toronto, ON, Canada; ^6^ Division of Orthopedic Surgery, Department of Surgery, University of Toronto, Toronto, ON, Canada

**Keywords:** osteoarthritis, DMM, CD200R1, mouse model, aptamer

## Abstract

Post-traumatic knee osteoarthritis is characterized by cartilage degeneration, subchondral bone remodeling, osteophyte formation, and synovial changes. Therapeutic targeting of inflammatory activity in the knee immediately post injury may alter the course of osteoarthritis development. This study aimed to determine whether CD200R1 agonists, namely the protein therapeutic CD200Fc or the synthetic DNA aptamer CCS13, both known to act as anti-inflammatory agents, are able to delay the pathogenesis of injury-associated knee osteoarthritis in a murine model. Ten week old male C57BL/6 mice were randomized and surgical destabilization of the medial meniscus (DMM) to induce knee arthritis or sham surgery as a control were performed. CCS13 was evaluated as a therapeutic treatment along with CD200Fc and a phosphate-buffered saline vehicle control. Oligonucleotides were injected intra-articularly beginning one week after surgery, with a total of six injections administered prior to sacrifice at 12 weeks post-surgery. Histopathological assessment was used as the primary outcome measure to assess cartilage and synovial changes, while µCT imaging was used to compare the changes to the subchondral bone between untreated and treated arthritic groups. We did not find any attenuation of cartilage degeneration or synovitis in DMM mice with CD200Fc or CCS13 at 12 weeks post-surgery, nor stereological differences in the properties of subchondral bone. The use of CD200R1 agonists to blunt the inflammatory response in the knee are insufficient to prevent disease progression in the mouse DMM model of OA without anatomical restoration of the normal joint biomechanics.

## Introduction

Post-traumatic knee osteoarthritis is a prevalent musculoskeletal disease leading to significant disability with enormous medical and socioeconomic consequences. It is characterized by articular cartilage degeneration, subchondral bone remodeling, osteophyte formation, and synovial changes, including inflammation.

Arthritic changes begin in the acute phase post-insult and progress ominously to symptomatic osteoarthritis (1, 2). With the goal of reducing the risk of osteoarthritis, surgery to optimize anatomic repair of the joint when possible is the primary management option in traumatic knee injuries. However, eligibility criteria for surgical repair are not well defined and depend on the nature of the injury and patient characteristics. Moreover, a large proportion of joint injuries are of lower severity and managed conservatively. The armamentarium of non-surgical treatments in such cases focuses on providing symptomatic relief and restoring joint mobility, critically leaving the need for arthritic management unmet (3). Therapeutics that can successfully halt the destructive inflammatory activity in the knee immediately after injury represents an ideal non-surgical option to alter the course of osteoarthritis development.

DNA aptamers are oligonucleotides that have shown promise in many applications as both therapeutic and imaging agents to treat patients with inflammatory diseases (4, 5). Aptamers can be developed against any biological targets and thus the resulting agents can be designed to specifically target or modulate key biological processes in humans and animals towards either dampening or augmenting inflammatory responses. Applications for these synthetic aptamers may be potentially found in reducing arthritis symptoms through specifically targeting clinically important immune molecules such as CD200R1, TNFα, and PD-1 (5).

CD200R1 is expressed on the surface of myeloid and lymphoid cells and delivers immune inhibitory signals to modulate inflammation when engaged with its ligand CD200 (6). As such signaling through the CD200R1 pathway plays a prominent role in limiting inflammation in a wide range of immune-related diseases and autoimmune disorders (including rheumatoid arthritis) by increasing the macrophage fusion interaction (formation of osteoclasts) to potentiate bone resorption (6). Our team recently developed a synthetic, pegylated aptamer (pegylated CCS13, referred to herein as CCS13) that acts as an agonist for the inhibitory immune checkpoint receptor CD200R1 (7). The consequence of the CCS13 aptamer binding to CD200R1 present on immune cells is to dampen the production of inflammatory cytokines, reducing the damaging immune responses that typically cause inflammation. This aptamer recognizes both human and mouse CD200R1 and thus can be evaluated in murine models of inflammation, including osteoarthritis.

**Figure 1:**
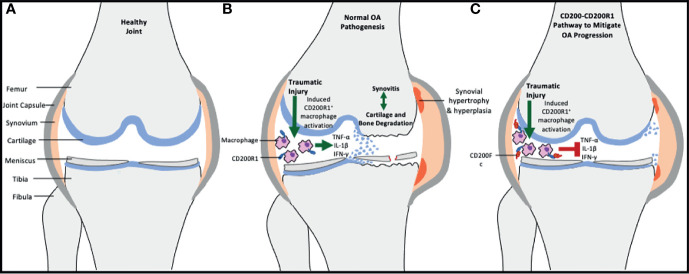
Schematic showing the role of CD200-CD200R1 pathway in osteoarthritis development. **(A)** demonstrates the anatomic structure of a healthy, non-arthritic joint. **(B)** In case of knee traumatic injury, a vicious cycle of reparative processes through inflammatory activity and structural damage is induced, leading to osteoarthritic changes. CD200R1 expressing macrophages are important mediators of synovitis through secretion of pro-inflammatory cytokines. **(C)** By upregulating CD200 expression (via exogenous CD200Fc or CCS13 aptamer), the CD200R1 positive macrophages are inhibited *via* the binding of CD200 to the receptor sites, hence leading to downregulation of proinflammatory cytokines and disruption of the vicious cycle.

The objective of this work was to determine whether the CD200R1 agonist aptamer CCS13 or a natural dimeric form of the CD200R1 ligand, namely, CD200Fc can delay the pathogenesis of injury-associated osteoarthritis in a murine model. It was hypothesized that this synthetic oligonucleotide may help reduce the progression of osteoarthritis pathologies including cartilage degeneration, synovitis, and bone remodeling (**Figure 1**).

## Methods

### Animal Model

All animal experiments were approved by the Animal Care Committee at Krembil Research Institute. Ten week old male C57BL/6 mice were randomized to destabilization of the medial meniscus (DMM), or sham surgery as a control, as previously described (8). Briefly, right hind limbs were sterilized and a 3mm longitudinal incision was made from distal patella to proximal tibial plateau and the medial meniscotibial ligament was identified. For the DMM group, this ligament was transected, and the destabilization of meniscal tissue was confirmed by lifting the free end of the meniscus. Transection was not performed for sham surgery. The soft tissues and overlaying skin were sutured. Mice were allowed unrestricted access to food and water and were fully weight-bearing on both hind limbs post-surgery. There were six groups (n=8/group) in this study: DMM+PBS, DMM+CCS13, DMM+CD200Fc, SHAM+PBS, SHAM+CCS13, and SHAM+CD200Fc.

### Treatment

**Figure 2:**
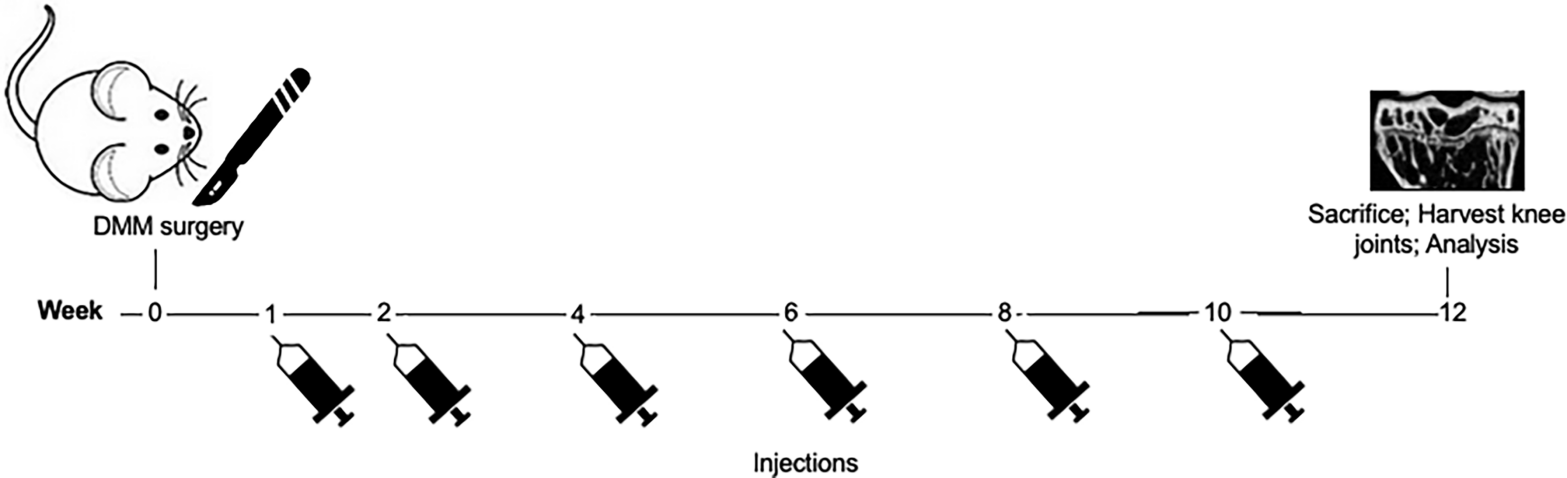
Schematic showing the protocol for treatment following DMM surgery. Knee surgery (sham or destabilization of medial meniscus) was performed on day 0. Intra-articular injections of either PBS (control), CD200Fc, or CCS13 were given in the healing joint at weeks 1, 2, 4, 6, 8, and 10. All mice were sacrificed at week 12 and the healing knee joints were analyzed *via* histology and microCT imaging.

CCS13 was evaluated as the therapeutic along with CD200Fc and phosphate-buffered saline (PBS; vehicle control). The formulation of CD200Fc and pegylated CCS13 (referred to as CCS13) is described elsewhere (7, 9). PBS or molarity-matched pegylated CCS13 (650 pmol) or CD200Fc (325 pmol) were administered *via* intra-articular injections, (3 microliters each) in the operated knee joints. Injections were started one week after surgery, with six injections administered over the course of the experimental period (**Figure 2**). All mice were sacrificed at 12 weeks post-surgery. Right hind limbs were harvested and immersed in 70% alcohol for fixing the tissue prior to imaging.

### Micro-CT Based Stereology

Harvested limbs were vertically aligned and scanned at 10 µm isotropic voxel size using energy settings of 55 kV, 200 µA, and beam hardening correction factor of 1200 mg hydroxyapatite (mgHA) per cm^3^ (µCT 100 scanner, Scanco Medical, Brϋttisellen, Switzerland). Reconstructed scans were exported as DICOM images and processed in AmiraDEV 5.3.3 (Visualization Sciences Group, FEI). The scans were cropped to bind the proximal tibia extending from the tibial plateau to the proximal tibial growth plate in 3D view. Next, the subchondral trabecular bone was manually segmented from 2D slices in the coronal plane. The resulting 3D ROI was divided through the depression between the intercondylar tubercles into lateral and medial condyles for separate evaluation. Stereological measures of bone volume (BV), total volume (TV), bone volume fraction (BV/TV), bone mineral density (BMD), and bone mineral content (BMC) were computed using CT Analyser software (SkyScan, Kontich, Belgium).

### Histology

After CT scanning, the limbs were stored in 70% alcohol until further processing. The specimens were fixed in TissuFix (Chaptec) overnight, decalcified in RDO Rapid Decalcifier (Apex Engineering) for 1.5 hours, refixed in TissuFix overnight, embedded in paraffin, and sectioned. Representative sections from the medial femur and tibia were stained with Safranin O Fast Green (Millipore Sigma) to analyze cartilage degradation (orange-red = proteoglycans, green = collagen/cytoplasm, black = nuclei) and Masson’s trichrome (blue = collagen, red = cytoplasm, black = nuclei) to analyze synovitis from 0 (normal) to 3 (severe) using OARSI scoring recommendations for mice (10). The cartilage on the femoral condyle and tibial plateau was scored separately from 0 (normal) to 6 (erosion >75% of articular surface) using the OARSI scoring system. Synovitis was scored from 0 (no synovitis) to 3 (severe synovitis) considering collagen deposition, cell infiltration, tissue thickness and tissue invasion onto the cartilage surface.

### Statistical Analysis

Mean values were compared for the primary outcome measures of synovitis and tibial plateau scores using one-way analysis of variance test (SPSS V18, IBM, IL, USA) between the six groups. One-way ANOVA and Student’s T-test with Sidak correction was used to compare stereological measures between groups. Results are reported as mean ± standard deviation. For all analyses, p<0.05 was considered significant.

## Results

No mice were lost during the experimental period. Two animals, one from SHAM+CCS13 and the second from SHAM+PBS, were excluded from all analysis as the specimens were damaged during the harvest procedure.

### Histology

**Figure 3:**
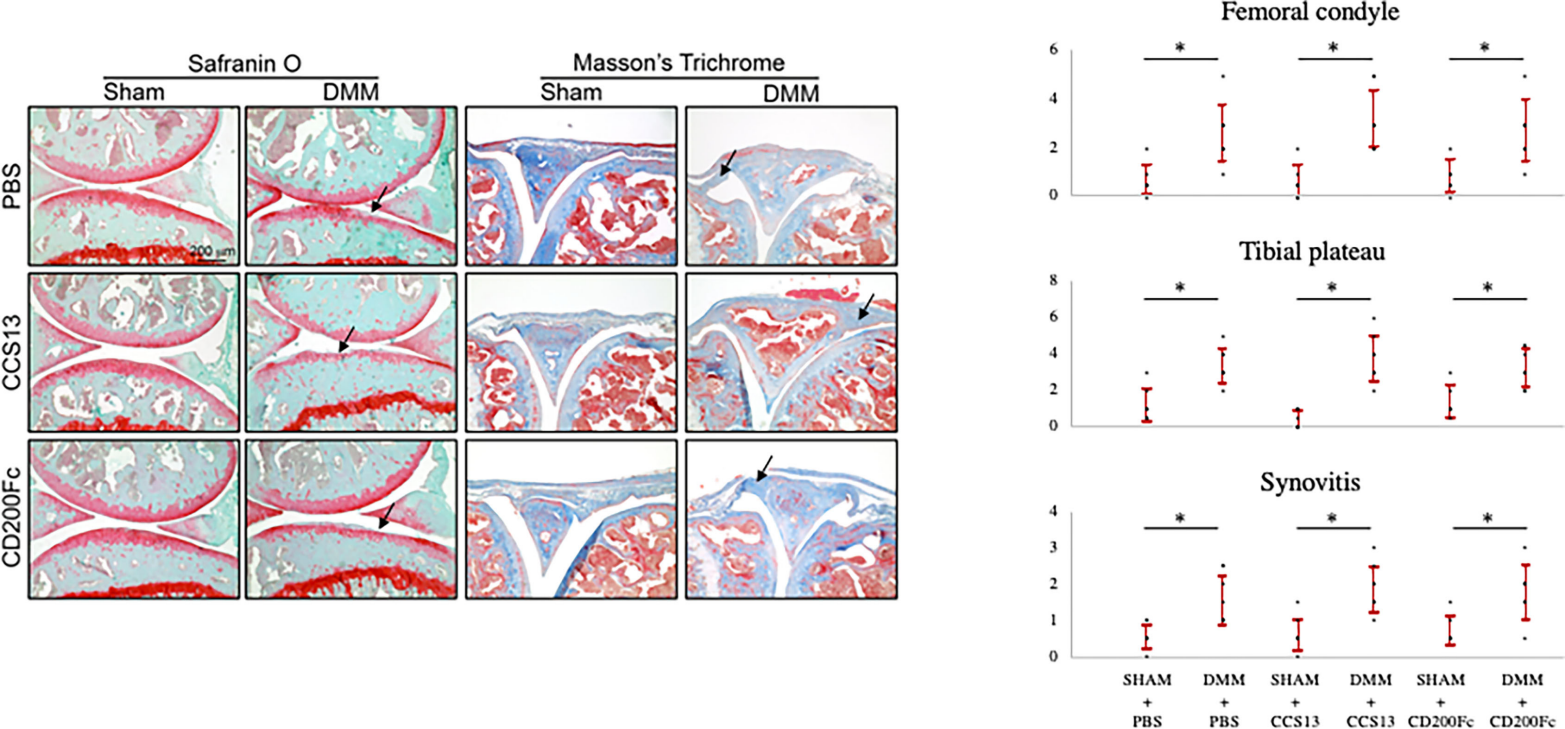
Histology images and scores of representative stained knee sections. ^1^The extent of cartilage loss in the femoral condyle and tibial plateau was scored separately using the OARSI scoring system: 0 [normal] to 6[erosion>75% of articular surface]. Synovitis was scored from 0 (normal) to 3 (severe). Safranin O-stained sections were used to score cartilage degeneration and Masson’s trichrome-stained sections were used to measure synovitis. Black arrows indicate areas of cartilage degeneration and synovitis in Safranin O or Masson’s Trichome-stained sections respectively. ^2^Dots represent individual data points and error bar represents standard deviation. ^3^Statistical analysis was conducted using one-way analysis of variance test to compare the six groups. *p < 0.05.

Average OARSI scores from safranin O-stained sections as a measure of cartilage degradation in the DMM+PBS group were 2.63 ± 1.19 for the femoral condyle and 3.38 ± 0.92 for the tibial plateau, which was indicative of moderate arthritis (**Figure 3**). These were significantly higher compared to average scores for SHAM+PBS group (healthy control; femoral condyle: 0.69 ± 0.59, p<0.01; tibial plateau: 1.19 ± 0.92, p<0.01). CCS13- treated DMM group had similar OARSI cartilage degeneration scores (femoral condyle:3.25 ± 1.16; tibial plateau: 3.75 ± 1.28) to the DMM+PBS group (femoral condyle:2.63 ± 1.19, p=0.31; tibial plateau: 3.38 ± 0.92, p=0.51). Moderate synovitis, as indicated by synovitis scores, was observed in all DMM groups (**Figure 3**). These data suggest that the dosing regimen of CCS13 in this does not attenuate mouse DMM-induced OA.

### Bone Stereology

**Figure 4:**
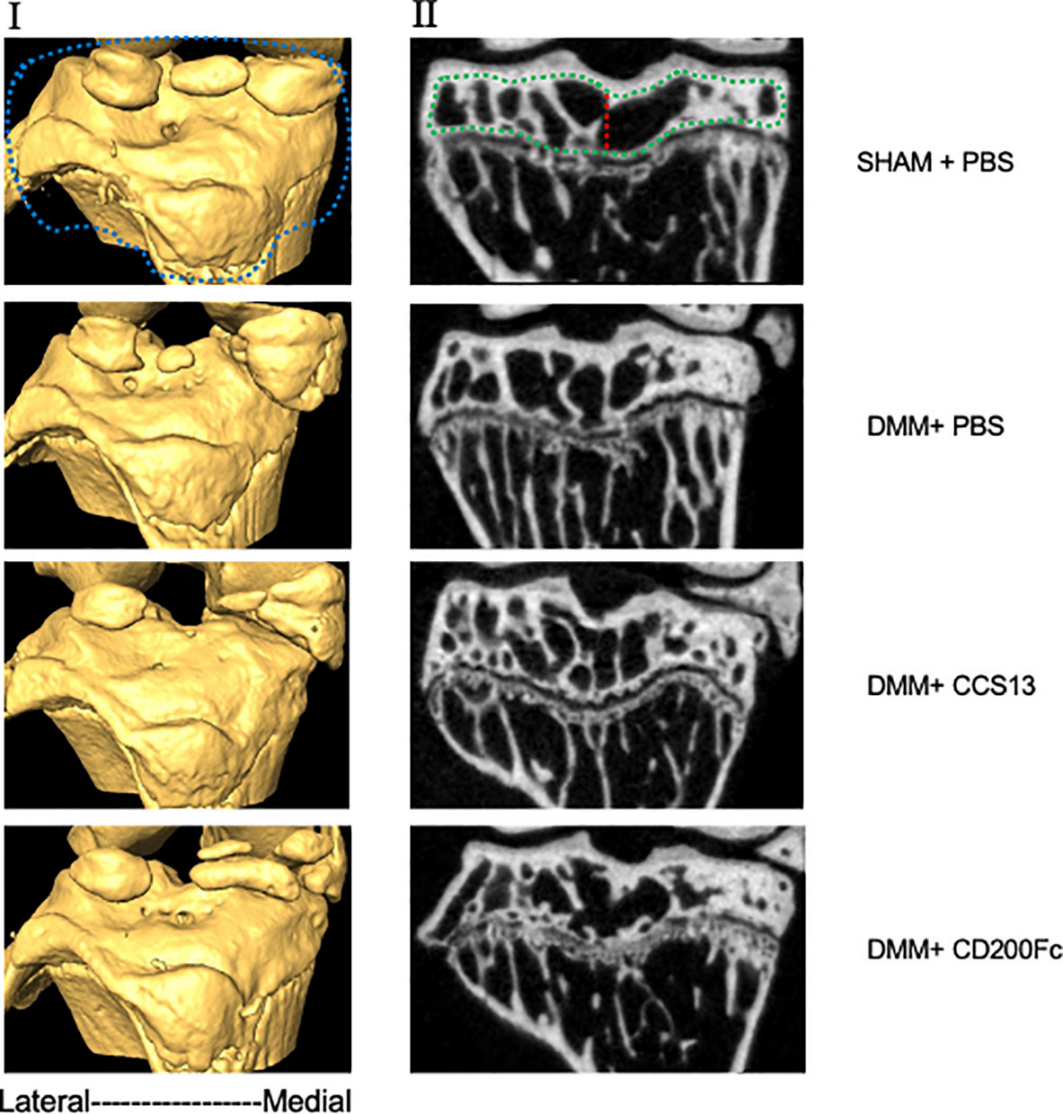
MicroCT images of representative specimens (3D model in panel I and 2D coronal slices in panel II). Stereological analysis involved a series of cropping steps in order to isolate the region of interest. First, the epiphyseal region, demarcated by blue line, was crudely selected in the 3D reconstructed model. Next, the subchondral trabecular bone (green line) was segmented in 2D slices. Finally, the segmented slices were merged and divided into lateral and medial halves (red line) and analyzed separately.

**Figure 4A** shows 3D models with coronal slices of knee joints at 12 weeks post-injury and **Figure 4B** shows means and standard deviations for stereological parameters at this timepoint. SHAM+CD200Fc had higher average total volume of subchondral bone compared to SHAM+PBS for lateral tibia (difference: 0.12 ± 0.04cm^3^; p=0.07) and medial tibia (difference: 0.21 ± 0.05cm^3^; p<0.01) indicating effect of CD200Fc injectable on bone tissue. For lateral tibia, DMM+PBS group had significantly higher average total volume compared to the SHAM+PBS group (difference: 0.14 ± 0.01cm^3^, p=0.01), however, the two groups had similar bone volume (difference: 0.04 ± 0.02cm^3^; p=0.09) and bone volume fraction (difference: -1.59 ± 0.95%, p=0.43). For medial tibia, DMM+PBS was similar to SHAM+PBS in bone properties. Compared to the DMM+PBS group, DMM+CCS13 had significantly higher bone volume (difference: 0.12 ± 0.03cm^3^, p=0.01) and total volume (difference: 0.18 ± 0.05cm^3^, p=0.02) but similar bone volume fraction (difference: 1.80 ± 2.43%, p=1). In comparison to SHAM+CD200Fc, DMM+CD200Fc group had significantly lower total volume (difference: -0.16 ± 0.04cm^3^, p=0.01), higher bone volume fraction (difference: 7.32 ± 0.59%,p<0.01), and higher BMD (difference:13.69 ± 0.79mgHA/cm^3^, p<0.01). No significant differences were seen between DMM+SHAM and DMM+CD200Fc or DMM+SHAM and DMM+CCS13 (**Figure 4B**).

## Discussion

To our knowledge, this is the first study to assess the therapeutic potential of the CD200-CD200R1 pathway in osteoarthritis. We evaluated the effect of upregulating CD200Fc and CD200Fc-based aptamer, CCS13, in a meniscal tear model. We utilized histopathology as the primary outcome measure to assess soft tissue changes, and µCT imaging to compare the changes in the subchondral bone between untreated and treated arthritic groups. Our analyses did not find any maintenance of cartilage tissue integrity in the disease state with the dose and intra-articular injection regimen of CD200Fc or CCS13 12 weeks post-injury. Similar to some previous studies, there were no stereological differences in the properties of subchondral bone between SHAM+PBS and DMM+PBS groups at 12 weeks as well as no differences between the DMM+PBS and the treated DMM groups at this timepoint (11, 12).

The search for therapeutic targets in osteoarthritis has failed to yield clinically translatable drugs that effectively attenuates disease progression in patients. An important shortcoming in previous works, which this study aimed to overcome, was targeting specific cytokines to dampen inflammation-induced damage. Osteoarthritis is complex and the strategy of targeting singular pro-inflammatory markers (most notably IL-1β) to tackle the disease, at least systemically, has been recognized as ineffective (13). In contrast, upstream targets, like macrophages themselves, offers better opportunities to control cytokine release and cell signaling, and hence modulate the biochemical response in osteoarthritis (14, 15).

CD200Fc attenuates early arthritic changes in animal models of rheumatoid arthritis. In the collagen-induced rheumatoid arthritis model, CD200Fc treatment significantly lowers expression of pro-inflammatory cytokines (IL-1β and TNFα) and lowers disease severity at 10 days post disease induction (16, 17). Our study was based on positive findings in the rheumatoid models as the etiology of osteoarthritis is similarly driven, in part, by inflammatory activity. In osteoarthritis, the acute phase after an injury is marked by infiltration of monocytes and macrophages to respond to the cellular damage, followed by a sustained low-grade inflammation in response to, albeit also causing, cartilage thinning and bone surface erosion (17). As such, suppressing pro-inflammatory myeloid cells *via* the CD200-CD200R1 pathway presents an opportunity to break the cycle that causes joint destruction following injury.

The distinction between the pathophysiology of osteoarthritis and rheumatoid arthritis may explain why CD200 based treatment failed in the osteoarthritis model despite being successful in the rheumatoid model. Unlike rheumatoid arthritis, the process of osteoarthritis involves dysfunctional biomechanics due to loss of structural integrity in addition to the altered biochemical activity (18). When left unrepaired, abnormal joint loading translates to persistent micro-damage with joint use, which further aggravates tissue damage and immune response. Thus, anti-inflammatory treatment may be sufficient to break the autoimmune cycle in rheumatoid arthritis which has no inciting traumatic intra-articular internal derangements, but it may not be sufficient in arthritic conditions resulting from repetitive damage occurring with dysfunctional joint loading from injury. To assess the role of joint biomechanics in driving osteoarthritic changes, Heard et al. (19) used a sheep anterior cruciate ligament (ACL) tear model (19). Grafting was performed as the intervention to restore mechanical stability and disease burden in these animals was compared with sham surgery controls and healthy controls. Despite the repair, acute inflammation was noted at two weeks post-surgery and could not be rescued by surgical intervention. However, the levels of inflammatory cytokines were restored to that of healthy controls by 20 weeks, indicating that surgical management with repair of the tear to restore the normal joint stability can delay osteoarthritis by preventing ongoing micro-trauma.

The parameters for treatment were chosen to maximize the benefit from treatments while being mindful of the clinical translatability of the regimen. CD200R1 are found on macrophages and macrophages are highly expressed, albeit in a cyclic manner, during the entirety of osteoarthritis (15, 20). As such, we opted for repeated, local delivery of CD200R1 agonist to target the persistent inflammation driven by macrophages. The following paragraphs describe the rationale for the choices made with respect to the treatment regimen. We also highlight areas of improvement that may be considered in designing exploratory drug studies in osteoarthritis.

Treatment onset was one week after DMM surgery. As the joint space is 5-10ul in volumetric size, injecting 3ul of drug dose in a swollen, injured joint may incite further inflammation (21, 22). However, this treatment timing implied that the initial surge of macrophage activity, which occurs at day 0-1 post-injury, was not targeted. Whether starting the treatment earlier (to curb the acute pro-inflammatory activity) would have translated to a chronic delay in the development of arthritic changes is uncertain.

The maximal frequency for treatment doses was deemed to be one time per week. More frequent injections in an already damaged intra-articular region may negatively impact healing. From a clinical standpoint, high frequency of knee injections is not a feasible strategy due to cost and long-term adherence associated with the management of osteoarthritis. Modulating other aspects of the dosing regimen and timepoint of analysis may be considered in future exploration of CD200 based therapeutics for osteoarthritis. As well, utilizing larger animals with similar knee size as humans may represent a more relevant strategy when testing therapeutics requiring frequent (for instance, once a week) injections.

Only limited dosing regimens were explored in this study, as described above. Further work may consider different dosing regimens and their utilization in combination with surgical meniscal repair to assess the full potential of CD200R1 agonists in halting arthritic changes. An additional methodological limitation is that immunostaining with CD200R1 antibody was not performed in this study. Analyzing the expression of CD200R1-positive immune cells should be considered in future work particularly to better understand if alternate treatment regimens may be effective. Finally, the study design did not involve serial analysis at multiple time points. As such, temporary benefits, if any, in the acute phase following drug injection remain unknown. However, clinical translation of short-term gain that does not result in a long-term benefit or symptom delay is not relevant in the management of a chronic disease like osteoarthritis. Patients with a traumatic knee injury would benefit most from treatments that can delay osteoarthritis in the range of months to years.

Nearly 50% of patients with traumatic knee injury develop OA (23). There remains a significant need for strategies to reduce chronic post-traumatic inflammation that contributes to the degenerative process of osteoarthritis that afflicts millions of individuals. While the use of aptamer interventions to blunt the persistence of the inflammatory response may be effective, it appears that without anatomical restoration of the normal joint biomechanics, biochemical treatments alone are insufficient to prevent disease progression. Even with surgical treatment to correct traumatic joint injuries, while the onset of osteoarthritis may be delayed, it does not entirely prevent degenerative changes leading to symptomatic presentation. Therefore, in appreciating the significance of the synergistic role of biomechanics and biochemical activity in osteoarthritis, future work may be directed at exploring the CD200-CD200R1 pathway in the context of surgically repaired joints.

## Data Availability Statement

The raw data supporting the conclusions of this article will be made available by the authors, without undue reservation.

## Ethics Statement

The animal study was reviewed and approved by Animal Care Committee at Krembil Research Institute.

## Author Contributions

KV: Methodology, Experiments, Formal Analysis, Manuscript Writing, and Editing. AP: Methodology, Experiments, Formal Analysis. SN: Methodology, Experiments, Formal Analysis. JR: Methodology, Formal Analysis, and Manuscript Review and Editing. AH: Manuscript Writing and Editing. MK: Methodology, Manuscript Review and Editing, Funding Acquisition, and Supervision. JG: Funding Acquisition, Conceptualization, Methodology, Manuscript Review and Editing, and Supervision. CW: Funding Acquisition, Conceptualization, Methodology, Resource Provision, Manuscript Writing and Editing, and Supervision. DN: Funding Acquisition, Conceptualization, Methodology, Resource Provision, Manuscript Writing and Editing, Supervision, and Project Administration. All authors contributed to the article and approved the submitted version.

## Funding

Support for this study was provided by FED DEV Ontario, CIHR operating grants 125862 and 148556 (JG) with matching from D5Pharma (in kind) and the Holland Bone and Joint Collaborative Research Innovation Fund (DN and CW). Animal studies in part were supported by the Tier 1 Canada Research Chair Award (MK).

## Conflict of Interest

The authors declare that the research was conducted in the absence of any commercial or financial relationships that could be construed as a potential conflict of interest.

## Publisher’s Note

All claims expressed in this article are solely those of the authors and do not necessarily represent those of their affiliated organizations, or those of the publisher, the editors and the reviewers. Any product that may be evaluated in this article, or claim that may be made by its manufacturer, is not guaranteed or endorsed by the publisher.
